# Circulating MicroRNA-26a in Plasma and Its Potential Diagnostic Value in Gastric Cancer

**DOI:** 10.1371/journal.pone.0151345

**Published:** 2016-03-24

**Authors:** Xiaonan Qiu, Jinyue Zhang, Weihong Shi, Sang Liu, Meiyun Kang, Haiyan Chu, Dongmei Wu, Na Tong, Weida Gong, Guoquan Tao, Qinghong Zhao, Fulin Qiang, Haixia Zhu, Qin Wu, Meilin Wang, Zhengdong Zhang

**Affiliations:** 1 Department of Environmental Genomics, Jiangsu Key Lab of Cancer Biomarkers, Prevention and Treatment, Collaborative Innovation Center For Cancer Personalized Medicine, Nanjing Medical University, Nanjing, China; 2 Department of Genetic Toxicology, The Key Laboratory of Modern Toxicology of Ministry of Education, School of Public Health, Nanjing Medical University, Nanjing, China; 3 Department of Medical Technology, Yancheng Insititute of Health Sciences, Yancheng, China; 4 Department of General Surgery, Yixing Cancer Hospital, Yixing, China; 5 Department of General Surgery, Huai-An First People’s Hospital Affiliated to Nanjing Medical University, Huai-An, China; 6 Department of General Surgery, The Second Affiliated Hospital of Nanjing Medical University, Nanjing, China; 7 Core Laboratory, Nantong Tumor Hospital, Nantong, China; Peking Union Medical College Hospital, CHINA

## Abstract

**Background:**

In the past decades, a good deal of studies has provided the possibility of the circulating microRNAs (miRNAs) as noninvasive biomarkers for cancer diagnosis. The aim of our study was to detect the levels of circulating miRNAs in tissues and plasmas of gastric cancer (GC) patients and evaluate their diagnostic value.

**Methods:**

Tissue samples were collected from 85 GC patients. Plasma samples were collected from 285 GC patients and 285 matched controls. Differentially expressed miRNAs were filtered with by Agilent Human miRNA Microarray and TaqMan low density array (TLDA) with pooled samples, followed by the quantitative reverse transcription polymerase chain reaction (qRT-PCR) validation. Receiver operating characteristic (ROC) curves were structured to evaluate the diagnostic accuracy of the miRNAs. The plasma level of miR-26a in GC patients of different clinical stages was compared.

**Results:**

Four miRNAs (miR-26a, miR-142-3p, miR-148a, and miR-195) revealed coincidentally decreased levels in tissue and plasma of the GC patients compared with controls, and ROC curves were constructed to demonstrate that miR-26a had a highest area under the ROC curve (AUC) of 0.882. Furthermore, miR-26a was stably detected in the plasma of GC patients with different clinical characteristics.

**Conclusion:**

Plasma miR-26a may provide a novel and stable marker of gastric cancer.

## Introduction

Gastric cancer (GC) is one of the most common malignancies in the world. Although the overall incidence has declined in the recent years, it is the fourth most common malignancy (989,000 cases) and the second leading cause of cancer-related death (738,000 deaths) worldwide [1]. More than 70% of cases occur in developing countries [2]. In particular, advanced-stage patients still demonstrate extremely poor survival rates [3].To date, the diagnosis of GC is based on the clinical symptoms, and there are some test techniques applied such as endoscopy, and barium meal examination. However, all of these approaches have shown some defects at detection of GC. In addition, there are some serum tumor markers for instance carcinoembryonic antigen (CEA), carbohydrate antigen 19–9 (CA19-9) and carbohydrate antigen 72–4 (CA72-4), and there are some limitations of the sensitivity and specificity for GC screening [4]. Hence, we pay more attention on discovering novel, dependable and noninvasive biomarkers of GC.

MicroRNAs (miRNAs) are a class of short (20~25 nucleotides), noncoding single-stranded RNAs [5]. It is widely believed that miRNAs can participate in regulating gene expression, giving rise to mRNA silencing such as target cleavage, translational inhibition, and mRNA decay [6]. Accumulating evidence has indicated that miRNAs were involved in several pathophysiological processes and lead to fundamental changes in multiple diseases. Zhang *et al*. found that miR-26a induced endothelial apoptosis and indicated a therapeutic potential of miR-26a for atherosclerosis associated with apoptotic cell death [7]. Leonov *et al*. described inhibition of miR-132 resulted in high *AGO2* expression, which was involved in angiogenesis and inflammation during primary endothelial cell activation [8]. Numerous studies have indicated that the miRNAs play indispensable roles on tumorigenesis, acting either as oncogenes or tumor suppressor genes [9]. Several miRNAs have reported to be related to cancer by the miRNA expression profiles [10–12]. Unfortunately, the tissue samples are difficult to acquire. Detecting miRNAs in tissues is not realistic in clinical application.

Moreover, increasing evidence has demonstrated that circulating miRNAs are resistant to RNase digestion and stably detectable in plasma and serum with ease of measurements [13–14]. Previous studies have confirmed that the existence of miRNAs in blood and the expression associated with cancer, including large B-cell lymphoma, colorectal cancer, bladder cancer and breast cancer [15–18]. These studies collectively provide a basis for circulating miRNAs acting as novel biomarkers for cancer screening.

Many studies demonstrate the expression level of miRNA in plasma is not completely consistent with that in tumor. miR-378 declined significantly in GC tissue and several cell lines [19], but miR-378 were meaningfully up-regulated in GC patients’ serum [20]. As the same as miR-486, miR-486 may function as a tumor-suppressor miRNA in gastric tumor [21], which were overexpressed in the GC patients’ serum [22]. At the present, several serum/plasma miRNAs can distinguish from the GC patients with the controls, nevertheless this can’t represent the miRNA expression in tissue. If we can find out the miRNAs both differentially expressed in gastric cancer tissue and plasma, it should have more clinical significance.

To ascertain whether the miRNAs differentially expressed both in tissue and plasma, we used two microarrays to screen miRNAs. In the two validation stages, four candidate miRNAs were significantly down-regulated in the tumor tissues and plasma samples by qRT-PCR. Our study indicated that miR-26a was significantly decreased stably in plasma of gastric cancer patients and could distinguish gastric cancer patients from controls, with good sensitivity and specificity. It was predicted to be a promising circulating biomarker of gastric cancer in routine clinical diagnosis.

## Materials and Methods

### Study design and subjects

This study was authorized by the institutional review board of Nanjing Medical University, and each participant provided the signed written informed consent. All of the subjects were recruited at Yixin Cancer Hospital, Nantong Tumor Hospital, Huai-An First People’s Hospital and The Second Affiliated Hospital of Nanjing Medical University from March 2006 to May 2013 in Jiangsu province, China.

The tumor tissues and the normal tissues (located > 5 cm away from the tumor) were obtained from 55 GC patients undergoing surgery and snap-frozen in liquid nitrogen. The plasma samples were collected from 285 GC patients, and 285 sex- and age-matched plasma from the healthy individuals were collected as the controls. All patients were proven the histologically diagnosis of GC at initial diagnosis, and none of them had received therapeutic procedures, for instance chemotherapy or radiotherapy. We classified the tumor stage in accordance with the tumor-node-metastasis (TNM) staging system of the Union for International Cancer Control (UICC). The clinical characteristics of study participants are presented in Table 1.

**Table 1:** Clinical characteristics of subjects.

Variables	The paired tissue samples (n = 50)	The plasma samples		
		Cases(n = 280)	Controls(n = 280)	
	N (%)	N (%)	N (%)	*P*
Age (years)				
Mean ± SD	64.79 ± 11.28	63.27 ± 9.90	63.23 ± 9.88	0.977^a^
≤ 65 years	25 (50.0)	149 (53.2)	149 (53.2)	0.534^b^
> 65 years	25 (50.0)	131 (46.8)	131 (46.8)	
Gender				
Male	37 (74.0)	177 (63.2)	177 (63.2)	0.535^b^
Female	13 (26.0)	103 (36.8)	103 (36.8)	
Lauren’s types				
Intestinal	27 (54)	126 (45.0)		
Diffuse	22 (44)	149 (53.2)		
Missing	1 (2)	5 (1.8)		
Invasion depth				
T1	3 (6.0)	45 (16.1)		
T2	6 (12.0)	40 (14.3)		
T3	26 (52.0)	172 (61.4)		
T4	15 (30.0)	17 (6.1)		
Missing	-	6 (2.1)		
Lymph nodes metastasis				
No	13 (26.0)	97 (34.6)		
Yes	37 (74.0)	176 (62.9)		
Missing	-	7 (2.5)		
Distant metastasis				
No	38 (76.0)	263 (93.9)		
Yes	12 (24.0)	14 (5)		
Missing	-	3 (1.1)		
TNM stage				
I	6 (12.0)	65 (23.2)		
II	13 (26.0)	61 (21.8)		
III	17 (34.0)	135 (48.2)		
IV	14 (28.0)	16 (5.7)		
Missing	-	3 (1.1)		

^a^ Student’s t test

^b^ Two-sided χ^2^ test

**Fig 1:**
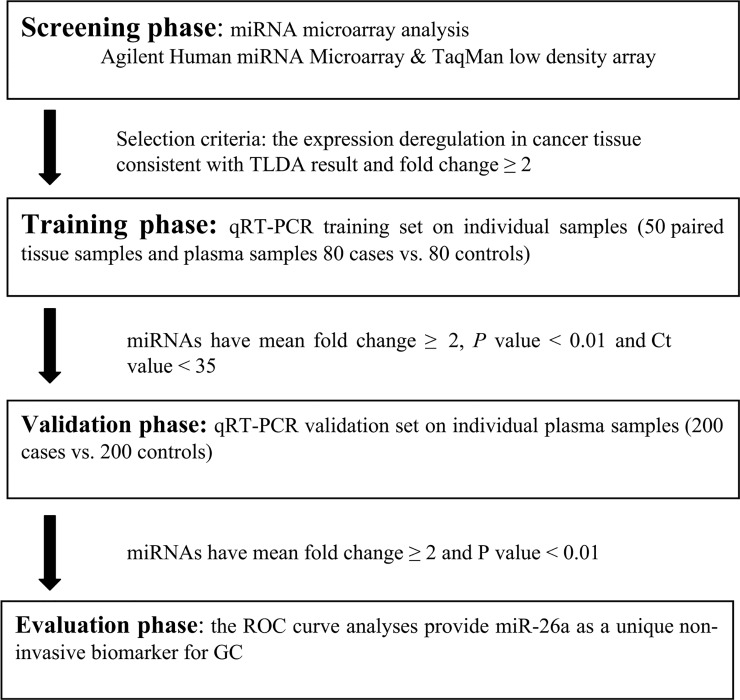
A flow chart of the experimental design.

A multi-stage study was designed to identify the plasma miRNAs as the biomarkers for GC patients (Fig 1). In the discovery stage, differentially expressed miRNAs were assessed in the paired tissue samples from 5 GC patients (S1 Table) by Agilent Human miRNA Microarray (V12.0, Agilent Technology, CA, USA). Plasma samples from 5 GC patients (S2 Table) whose sex, age clinical states matched with the tissue samples and 5 healthy controls were subjected to TLDA (V2.0; Applied Biosystems, Foster city, CA, USA) to identify the differentially expressed miRNAs. In the training phase, the significantly altered miRNAs were first evaluated by qRT-PCR in 50 paired tissues samples and 80 GC serum samples and 80 matched controls. Subsequently, the differentially expressed miRNAs between the tissue and serum were further examined in additional 400 participants, which included 200 GC patients and 200 controls.

### RNA isolation from plasma and tissue

Blood samples in the separate vacuum cubes were centrifuged at 3,000 rpm for 10 min at 4°C. We collected the supernatant and stored them at -80°C. Before starting the isolation procedure, we added a final concentration of 10^−4^ pmol/μl of synthetic *C*. *elegans* miR-39 (cel-miR-39) (Takara, Japan) to each sample, in order to controlling the biological variability. The RNA was extracted from 100 μl of plasma using QIAGEN miRNeasy Mini Kit (Qiagen, Valencia, CA, USA). The total RNA of tissues was isolated using TRIZOL reagent (Invitrogen, Carlsbad, CA, USA).

### Microarrays and qRT-PCR

Profiling was carried out using Agilent Human miRNA Microarray (V12.0, Agilent Technology, CA, USA) and TaqMan low-density array (V2.0; Applied Biosystems, Foster City, CA, USA). For the total RNA isolating from tissues, qRT-PCR was performed using SYBR(R) PrimeScript™ RT-PCR Kit (Takara Bio). A detailed description of the experimental protocols is deposited in the S1 Data.

### Statistical analyses

The paired tissue samples were compared using a Wilcoxon rank sum test. Non-parametric Mann-Whitney U test was performed to determine the significance in plasma miRNA levels between the cancer group and healthy group. Receiver-operator characteristic (ROC) curves and the areas under the ROC curves (AUCs) were obtained to assess the GC detection potential of miRNAs. Statistical analyses were two-sided test, and *P* < 0.05 was considered statistically significant. All the statistical analyses were performed with SPSS software version 16.0 (SPSS Inc., Chicago, IL, USA).

## Results

### Discovery of candidate biomarkers by microarrays

We used two microarray platforms for the miRNAs to analyze and compare the miRNA expression patterns from two different conditions. TLDA analysis was completed to confirm candidate miRNAs expression levels. A total of 142 miRNAs were detected in plasma. 65 of the total miRNAs were increased and 77 were decreased in GC patients compared with controls.

To identify candidate miRNAs, the expression of miRNAs between patients and controls were necessary to correspond with two criteria: (1) the expression deregulation in tumor tissue was consistent with TLDA outcome[23]; and (2) the plasma miRNA levels proving ≥ 2-fold difference between patients and controls. Ultimately, four down-regulated plasma miRNAs (i.e., miR-26a, miR-142-3p, miR-148a and miR-195) were selected as the candidates for the fist-stage validation (Table 2).

**Table 2:** The comparison of the partly results of microarrays.

miRNAs	Agilent Human miRNA Microarray		TaqMan low density array		
	Ct	Fold change	CasesΔCt^a^	ControlsΔCt	ΔΔCt^b^
hsa-miR-99a	-	2.034623	11.60988	13.53345	-1.92357
hsa-miR-17	-	3.120369	2.98488	4.20745	-1.22257
hsa-miR-199a-3p	-	2.066154	8.43588	9.13345	-0.69757
hsa-miR-483-5p	-	2.006861	7.67088	8.21245	-0.54157
hsa-miR-18a	-	4.948928	9.67688	10.13345	-0.45657
hsa-miR-27b	-	0.628604	12.52188	12.09045	0.43143
hsa-miR-145	-	0.6572	10.60988	9.88745	0.72243
hsa-miR-375	-	0.333354	12.18088	11.02345	1.15743
hsa-miR-215	-	0.470013	13.95988	12.30445	1.65543
**hsa-miR-148a**	-	0.564722	11.02288	8.98245	**2.04043**
**hsa-miR-142-3p**	-	0.577768	9.70188	7.58945	**2.11243**
**hsa-miR-26a**	-	0.679287	10.71788	7.95645	**2.76143**
**hsa-miR-195**	-	0.562931	14.94188	10.08245	**4.85943**

^a^ΔCt = Ct_sample_—Ct_cel-39_

^b^ΔΔCt = ΔCt_case_ -ΔCt_control_

### Confirmation of miRNAs by qRT-PCR

**Fig 2:**
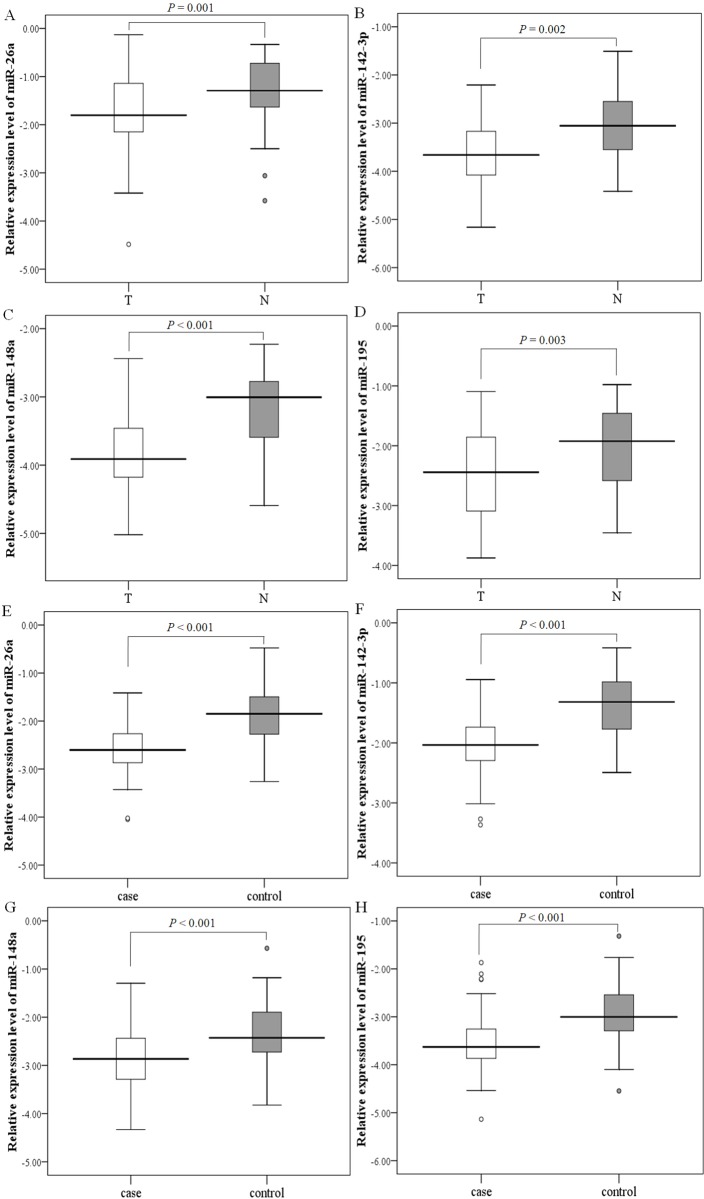
Box plots of relative expression levels of miRNAs by qRT-PCR in the training phases. (A-D) Relative expression levels of miR-26a, miR-142-3p, miR-148a, miR-195, in 50 paired gastric cancer tissues and corresponding noncancerous tissues (log_10_ scale on Y -axis). (E-H) Plasma expression levels of miR-26a, miR-142-3p, miR-148a, miR-195, in 80 GC patients and 80 healthy controls. The lines inside the boxes denote the medians (log_10_ scale on Y -axis). The whiskers of box plots: Min to Max.

Firstly, the results of microarrays analyses were confirmed using the paired tissues, the GC plasma and the matched controls for these four miRNAs by qRT-PCR. Our results demonstrated that the tendency of the results of qRT-PCR was similar to the microarrays analyses, either in tissues or in plasma. The plot showed that the expression levels of four miRNAs were statistically significant in the GC tissues (*P* = 0.001, *P* = 0.002, *P* < 0.001 and *P* = 0.003 for miR-26a, miR-142-3p, miR-148a and miR-195, respectively, Figs 2A–2D). In the meantime, we performed qRT-PCR to verify the expressions of miRNAs were down-regulated in another independent set of 80 GC plasma samples and 80 healthy controls. The expressions of four miRNAs in GC plasma were significantly decreased compared to those in controls (all *P* < 0.001, Figs 2E–2H). The expressions levels of the four miRNAs in plasma were consistently decreased, comparing with tissue samples.

### Large-scale validation of the plasma microRNAs in GC patients

**Fig 3:**
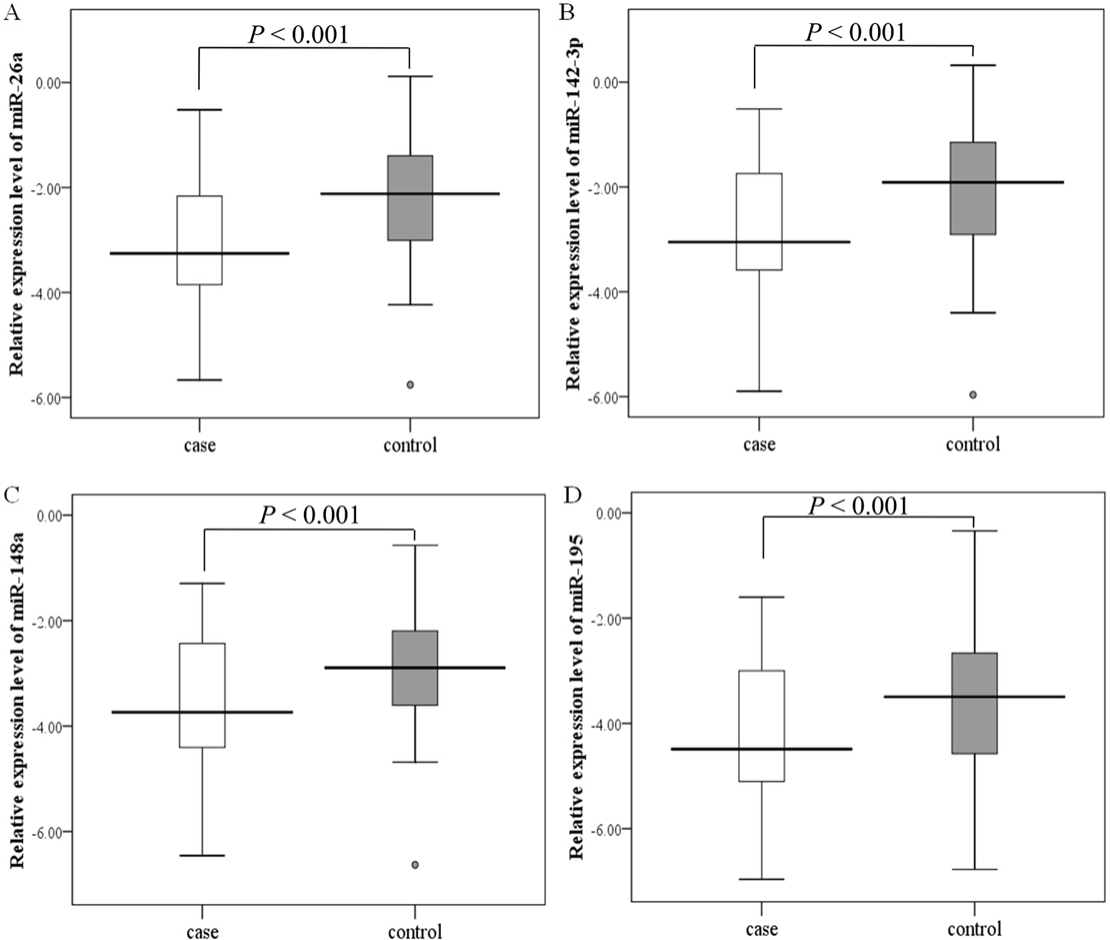
Plasma levels of four miRNAs in the validation phase. Box plots showed the plasma levels of miR-26a (A), miR-142-3p (B), miR-148a (C) and miR-195 (D) in 200 gastric cancer patients and 200 age- and gender-matched healthy controls. The y axis represents relative expression of miRNAs normalized to cel-miR-39 (Log_10_ relative level).

In order to validate stability of the plasma expressions of miRNAs, we performed qRT-PCR in a large scale of 200 GC plasma samples and 200 matched cancer-free controls. Accordant with the results of the training phase, the expression levels of four miRNAs showed significant down-regulation in plasma of GC patients (all *P* < 0.001, Fig 3), suggesting that these four miRNAs in plasma may serve as the candidate biomarkers for diagnosis of GC.

### Evaluation of the diagnostic potential of miRNAs for GC

**Fig 4:**
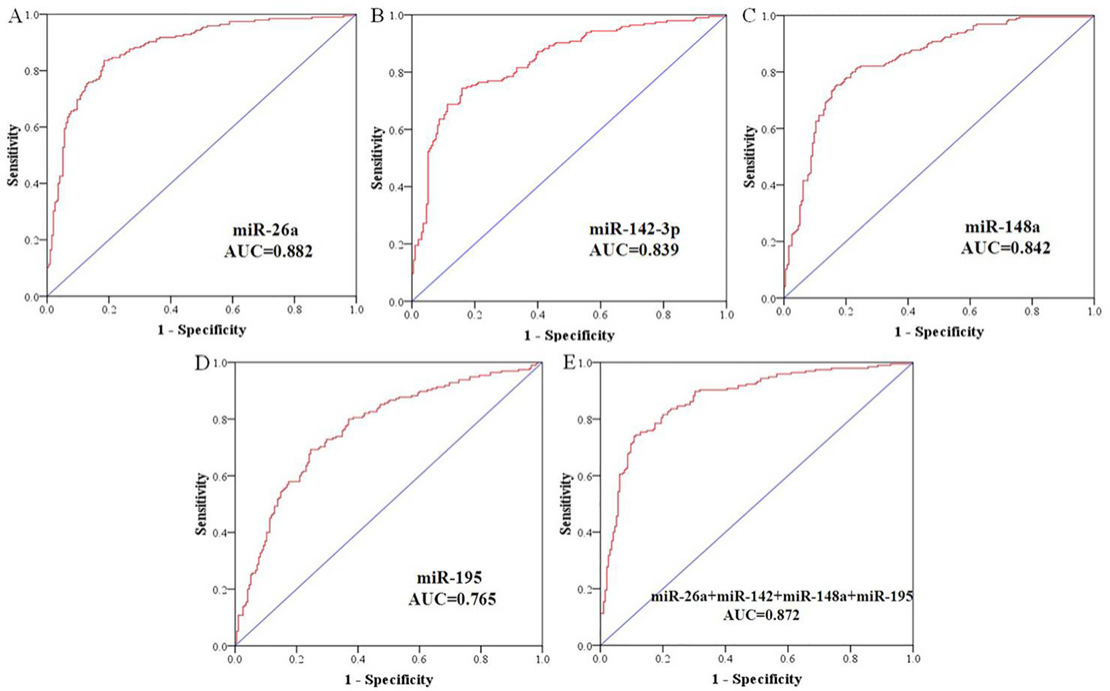
ROC curve analysis using four miRNAs for discriminating GC patients from healthy controls. ROC curves were constructed to show AUCs of miR-26a (A), miR-142-3p (B), miR-148a (C), miR-195 (D) and the combination of four miRNAs (E).

To assess the efficiency of above four miRNAs as diagnostic markers for GC detection, we performed ROC curve analysis on each miRNA. Fig 4 revealed that the ROC curves of four candidate miRNAs demonstrated the plasma miRNAs were of value to distinguish GC patients from normal controls. The cutoff value of 0.651 was equal to sensitivity + specificity-1 which was maximal for miR-26a (relative expression normalized by cell-39). For miR-26a, the sensitivity was 83.6% and the specificity was 81.5% with an AUC of 0.882 (95% CI = 0.847–0.916) (Fig 4A). At the cut-off value of 0.585 for miR-142-3p, the sensitivity was 74.4% and the specificity was 84.1% with an AUC of 0.839 (95% CI = 0.800–0.879) (Fig 4B). The AUCs were 0.842 (95% CI = 0.803–0.882) and 0.765 (95% CI = 0.717–0.812) for miR-148a and miR-195, respectively. The sensitivity and specificity were 75.4% and 83.1% for miR-148a, 69.2% and 75.4% for miR-195, respectively (Fig 4C and 4D). The combination ROC curve analysis yielded the AUC value of 0.872 (95% CI = 0.836–0.908) (Fig 4E).

To verify the diagnosis value of the host miRNA for GC, we combined any of the plasma miRNAs and performed the ROC curves as shown in S1 and S2 Figs. These data indicated that the miR-26a which showed the greatest ability for differentiating the GC patients from controls, could act as a suitable biomarker for detecting GC.

## Discussion

In spite of a significant decline in GC mortality in the past decade, the mortality rate is still higher than many other common malignancies [24]. In recent years, the tumor biomarkers for instance CEA, CA19-9, CA74-2 with the limited sensitivity and specificity have been widely used for diagnosis, monitoring and prognosis of GC [4]. All in all, there is of great significance for diagnosis of GC to look for more specific and sensitive biomarkers.

Tissue miRNAs as the biomarkers of diagnosis and prognosis were first proposed. Bandres *et al*. revealed that miR-451 was down-regulated in primary gastric and colorectal tumors, and the down-regulation of miR-451 was associated with low DFS and low overall survival [25]. Xiao *et al*. identified miR-106a as a dramatically up-regulated miRNA in GC [26]. They also found that the expression of miR-106a was significantly related to tumor stage, lymphatic, distant metastasis and invasion. Nonetheless, many types of cancer patients have been unable to undergo surgery in clinical practice when they are diagnosed. The reliance on surgical section and invasive procedure for tissue sample collection strongly limited the application of miRNAs in tissue of cancer diagnosis [27]. The diagnostic quality of these methods is unsatisfactory.

Emerging evidence also implies that circulating miRNAs are related to cancer [28–30]. The recent study reported that the miRNAs were detectable in plasma and stable at room temperature and/or multiple freezing-thawing processes [31]. Numerous reports had illuminated that the usefulness of circulating miRNAs as novel noninvasive biomarkers for cancers, such as colorectal cancer [32], breast cancer [33–34] and renal cell carcinoma [35].

In our study, we methodically screened the miRNA profile in the paired tissues and in plasma from GC patients and healthy controls. Plasma obtained from 5 cases and 5 controls were mixed together to form two pooled samples and were used for TLDA. This method of pooling samples is a widely used technique that has been shown to be reliable for profiling [36–38]. Comparing TLDA analysis and the result of tissue microarray, four miRNAs(miR-26a, miR-142-3p, miR-148a and miR-195) reduced in GC were selected.

Subsequently, we identified four candidate miRNAs by qRT-PCR in two stages, and found the expression levels of four miRNAs slumped in the plasma of GC patients which were in agreement with the expressions in tissues. ROC curve, also known as sensitivity curve, is widely recognized as an evaluation method of diagnostic test. It is also an integrated indicator reflecting the sensitivity and specificity of continuous variables. In the current study, ROC analysis indicated that the combination of four miRNAs wasn’t more effective than miR-26a individually. MiR-26a alone could achieve a good diagnostic efficiency in distinguishing GC patients from healthy controls with an AUC of 0.882 (sensitivity = 83.6% and specificity = 81.5%). A previous study by Schneider *et al*. displayed the serum levels of CEA, CA19-9 and CA72-4 in GC elucidated a sensitivity of 45.5% for CA19-9, 23.8% for CEA and 60.7% for CA72-4 [39]. These were far less than the sensitivity of miR-26a. Furthermore, the expression level of miR-26a in plasma was significant lower in the GC patients, and didn’t show any significance with the progression of GC (S3 Fig). Hence, miR-26a had great potential as a diagnostic plasma biomarker for GC.

In the past decades, the previous studies didn’t verify that whether the miRNA expression of plasma or serum could represent the levels of tissue. Compared with these studies, our study had own feature owing to reasons as follows. First, we selected the miRNAs which expressed differentially both in tissue and plasma, by comparing the results of two microarrays. This resulted in a better opportunity to identify feasible diagnostic markers. Furthermore, we found that the miR-26a expression significantly reduced in GC patients and kept stable in the plasma (S3 Fig).

The results revealed that the plasma expression of miR-26a was significant lower in the GC patients compared with the healthy controls (*P* < 0.05), which showed stability in GC patients with different clinical status. The down-regulation of plasma miR-26a expression may indicate the probability of diagnosing GC. Therefore, miR-26a may be a best choice to serve as potential biomarker for GC. Deng *et al*. showed that miR-26a levels in GC tissues were more remarkably decreased than those in non-tumor tissues by qRT-PCR [40]. They also demonstrated that miR-26a inhibited tumor growth and metastasis partly by targeting *FGF9* in vivo. These consisted with our results. These findings confirmed that miRNA-26a in plasma could be a potential diagnostic marker for GC.

However, the limitations of our study are that the samples for the tissue microarray and for the TLDA were not from the same patients, and our sample sizes for the microarrays were relative small. Further analysis of the microarrays for the same patients and the enlarged-sample confirmation are required to be applied as a novel screening tool for GC in the routine clinical utilization. Additionally, we didn’t choose the up-regulated miRNAs because of the fact that there were not the up-regulated miRNAs whose fold changes were ≥ 2. We selected candidate miRNAs which were profusely expressed in plasma to make sure of the probability of examination in clinical practice.

## Conclusions

In summary, our study revealed four down-regulated miRNAs, miR-148a, miR-142-3p, miR-26a, and miR-195, in GC patients. More importantly, the plasma expression of miR-26a was significantly reduced and stable in patients with gastric cancer. As a result, miR-26a may provide a non-invasive and stable biomarker of gastric cancer diagnosis and screening.

## Supporting Information

S1 Data Experimental Protocols of Microarrays and qRT-PCR. (DOC)

S1 Fig ROC curve analysis of the combination of two miRNAs among the four candidate miRNAs. The combination of miR-26a and miR-142-3p (A), the combination of miR-26a and miR-148a (B), the combination of miR-26a and miR-195 (C), and the combination of miR-142-3p and miR-148a (D), the combination of miR-142-3p and miR-195(E) and the combination of miR-148a and miR-195 (F) yielded the largest AUCs.
(DOC)

S2 Fig ROC curve analysis of the combination of three miRNAs among the four candidate miRNAs. The combination of miR-26a, miR-142-3p and miR-148a (A), the combination of miR-26a, miR-142-3p and miR-195 (B), the combination of miR-26a, miR-148a and miR-195 (C) and the combination of miR-142-3p, miR-148a and miR-195 (D) yielded the largest AUCs.
(DOCX)

S3 Fig Plasma level of miR-26a in gastric cancer patients stratified by the clinical status. Box plots showed the plasma levels of miR-26a in 200 healthy controls and 200 gastric cancer patients at different clinical status.
(DOCX)

S1 Table Clinical characteristics of tissue subjects screening by Agilent Human miRNA Microarray. (DOC)

S2 Table Clinical characteristics of plasma subjects screening by TLDA. (DOC)
