# Supplementary material for: Circulating MicroRNA-26a in Plasma and Its Potential Diagnostic Value in Gastric Cancer
Source: PLoS One. 2016 Mar 24;11(3):e0151345. doi: 10.1371/journal.pone.0151345 (PMC4806920; doi:10.1371/journal.pone.0151345)
Supplement: S1 Data — (DOC) [file pone.0151345.s001.doc]

**S1 Data. Experimental Protocols of Microarrays and qRT-PCR**

The cases for TLDA and the cases for tissue microarray were matched in age, sex, histopathological characteristics and tumor stage. The sex- and age-matched healthy subjects were collected as the plasma controls. We pooled the plasma samples with a fixed volume of 1,000 µl. After the total RNA extracting, the miRNAs were reverse transcribed using the TaqMan MicroRNA Reverse Transcription Kit and TaqMan MicroRNA Megaplex RT Human Pool Sets A & B (Applied Biosystems, Foster City, CA, USA). In order to increase the sensitivity of the TLDA, pre-amplification reactions were performed using the TaqMan PreAmp Mastermix and the Megaplex PreAmp Primer Pools A+B (Applied Biosystems, Foster City, CA, USA) according to the manufacturer’s protocol. qRT-PCR was carried out on an Applied Biosystems 7900HT thermocycler (Applied Biosystems, Foster City, CA, USA) using the cycling conditions recommended by the manufacturer. Raw data were exported using SDS software v2.3 (settings: automatic baseline; threshold, 0.2) and analyzed with the RQ manager software (Applied Biosystems, Foster City, CA, USA). The plasma miRNA levels were normalized relative to the cel-miR-39 and were calculated using the 2^-ΔCt^ method.

The plasma total RNA was reverse transcribed to complementary DNA by using the TaqMan MicroRNA Reverse Transcription Kit and miRNA-specific RT primers (Applied Biosystems). Real-time PCR reactions for the plasma samples comprised 2.5 µl TaqMan 2 × Universal PCR Mater Mix with No AmpErase UNG (Applied Biosystems , Germany), 0.25 µl 20 × miRNA-specific primer/probe mix (Applied Biosystems, Germany) and 2.25 µl of the reverse transcription product (diluted 1:10). Real-time PCR was carried out under the following conditions: 95 °C for 10 min, 40 cycles of 95 °C for 15 s and 60 °C for 1 min. Each sample was run in triplicate.

The miRNA levels of tissues were calculated based on the cycle passing threshold (Ct) values, normalized by U6 small nuclear RNA(RNU6B) expression, according to the equation 2^-ΔCt^.
