# Supplementary material for: Circulating MicroRNA-26a in Plasma and Its Potential Diagnostic Value in Gastric Cancer
Source: PLoS One. 2016 Mar 24;11(3):e0151345. doi: 10.1371/journal.pone.0151345 (PMC4806920; doi:10.1371/journal.pone.0151345)

**S2 Fig.** ROC curve analysis of the combination of three miRNAs among the four candidate miRNAs. The combination of miR-26a, miR-142-3p and miR-148a (A), the combination of miR-26a, miR-142-3p and miR-195 (B), the combination of miR-26a, miR-148a and miR-195 (C) and the combination of miR-142-3p, miR-148a and miR-195 (D) yielded the largest areas under the ROC curves (AUCs).


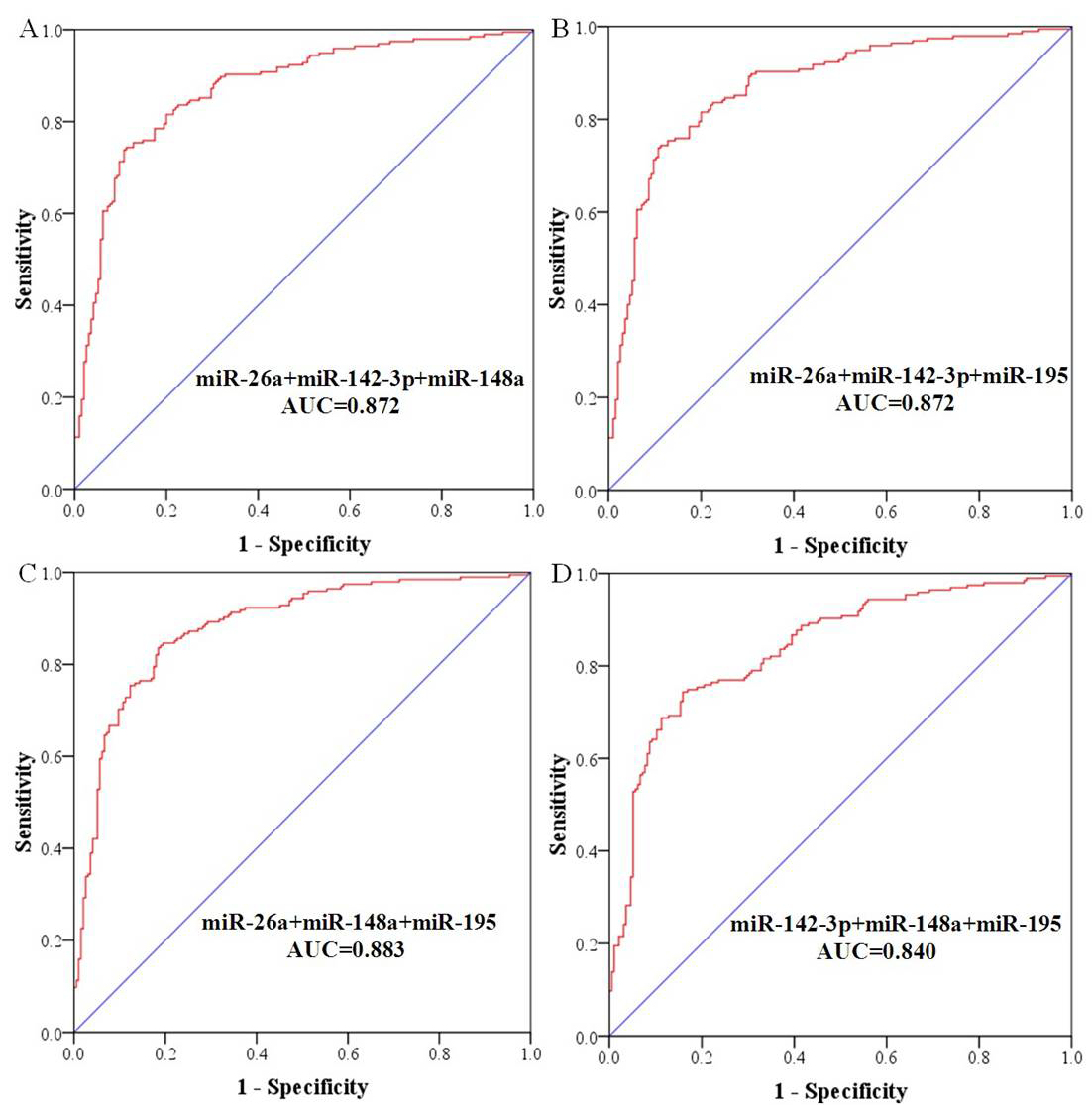

Supplement: S2 Fig — The combination of miR-26a, miR-142-3p and miR-148a (A), the combination of miR-26a, miR-142-3p and miR-195 (B), the combination of miR-26a, miR-148a and miR-195 (C) and the combination of miR-142-3p, miR-148a and miR-195 (D) yielded the largest AUCs. (DOCX) [file pone.0151345.s003.docx]
