# Supplementary material for: Circulating MicroRNA-26a in Plasma and Its Potential Diagnostic Value in Gastric Cancer
Source: PLoS One. 2016 Mar 24;11(3):e0151345. doi: 10.1371/journal.pone.0151345 (PMC4806920; doi:10.1371/journal.pone.0151345)

**S3 Fig.** Plasma level of miR-26a in gastric cancer patients stratified by the clinical status. Box plots showed the plasma levels of miR-26a in 200 healthy controls and 200 gastric cancer patients at different clinical status.


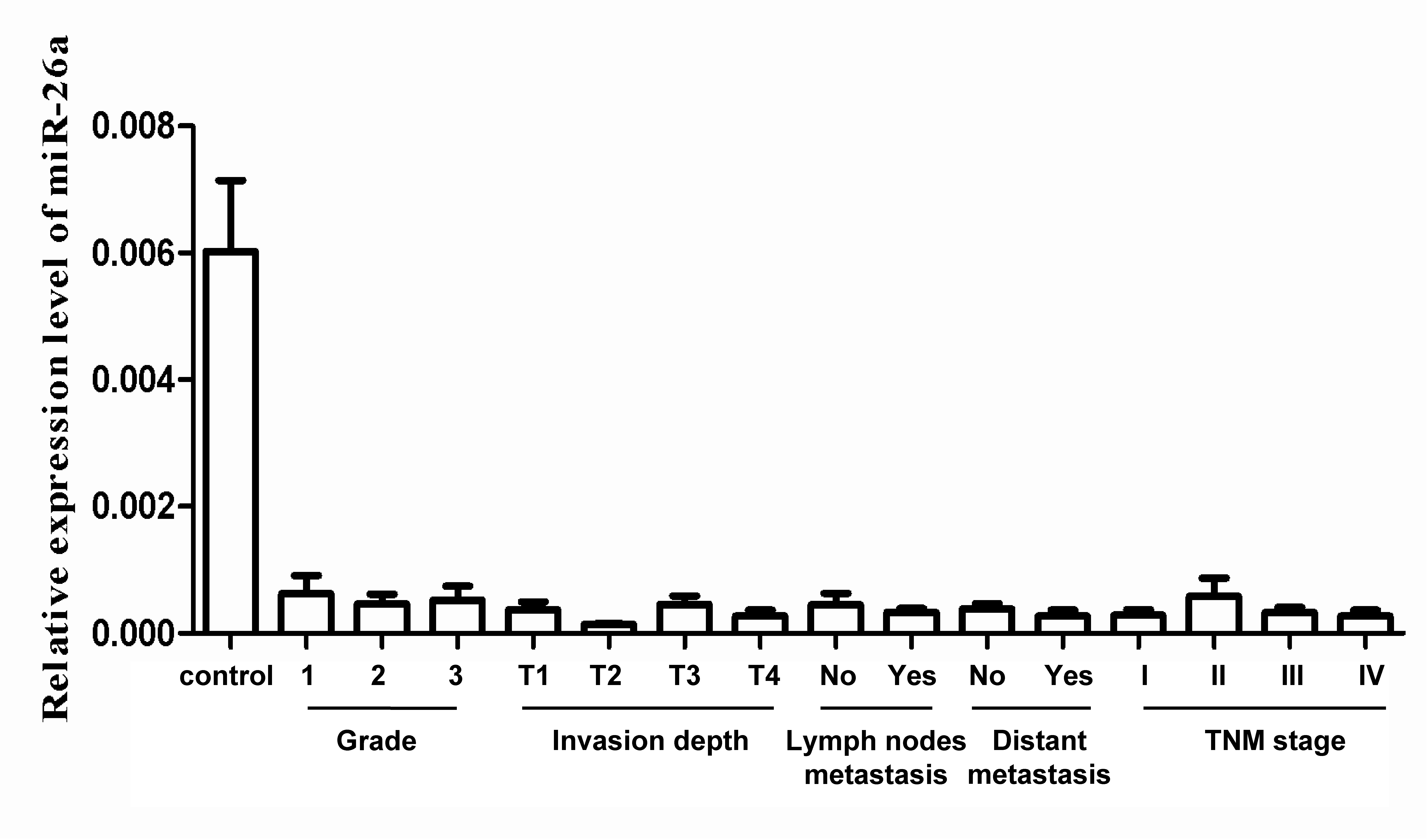

Supplement: S3 Fig — Box plots showed the plasma levels of miR-26a in 200 healthy controls and 200 gastric cancer patients at different clinical status. (DOCX) [file pone.0151345.s004.docx]
