# Supplementary material for: Circulating MicroRNA-26a in Plasma and Its Potential Diagnostic Value in Gastric Cancer
Source: PLoS One. 2016 Mar 24;11(3):e0151345. doi: 10.1371/journal.pone.0151345 (PMC4806920; doi:10.1371/journal.pone.0151345)
Supplement: S1 Table — (DOC) [file pone.0151345.s005.doc]

**S1 Table. Clinical characteristics of tissue subjects screening by Agilent Human miRNA Microarray**.

| No | Age(years) | Sex | TNM stage | Grade | Tumor site | Histological type |
| --- | --- | --- | --- | --- | --- | --- |
| Case 1 | 66 | Male | T3N1M0 | G2 | Cardia | Intestinal |
| Case 2 | 57 | Male | T2N0M0 | G3 | Cardia | Intestinal |
| Case 3 | 68 | Male | T3N0M0 | G2 | Cardia | Intestinal |
| Case 4 | 54 | Female | T3N0M0 | G3 | Cardia | Diffuse |
| Case 5 | 68 | Male | T2N0M0 | G2 | Cardia | Intestinal |
